# A Comprehensive Survey on Bone Segmentation Techniques in Knee Osteoarthritis Research: From Conventional Methods to Deep Learning

**DOI:** 10.3390/diagnostics12030611

**Published:** 2022-03-01

**Authors:** Sozan Mohammed Ahmed, Ramadhan J. Mstafa

**Affiliations:** 1 Department of Computer Science, Faculty of Science, University of Zakho, Duhok 42002, Iraq; sozan.ahmed@staff.uoz.edu.krd; 2 Department of Computer Science, College of Science, Nawroz University, Duhok 42001, Iraq

**Keywords:** knee osteoarthritis, bone segmentation, deep learning, segmentation, machine learning

## Abstract

Knee osteoarthritis (KOA) is a degenerative joint disease, which significantly affects middle-aged and elderly people. The majority of KOA is primarily based on hyaline cartilage change, according to medical images. However, technical bottlenecks such as noise, artifacts, and modality pose enormous challenges for an objective and efficient early diagnosis. Therefore, the correct prediction of arthritis is an essential step for effective diagnosis and the prevention of acute arthritis, where early diagnosis and treatment can assist to reduce the progression of KOA. However, predicting the development of KOA is a difficult and urgent problem that, if addressed, could accelerate the development of disease-modifying drugs, in turn helping to avoid millions of total joint replacement procedures each year. In knee joint research and clinical practice there are segmentation approaches that play a significant role in KOA diagnosis and categorization. In this paper, we seek to give an in-depth understanding of a wide range of the most recent methodologies for knee articular bone segmentation; segmentation methods allow the estimation of articular cartilage loss rate, which is utilized in clinical practice for assessing the disease progression and morphological change, ranging from traditional techniques to deep learning (DL)-based techniques. Moreover, the purpose of this work is to give researchers a general review of the currently available methodologies in the area. Therefore, it will help researchers who want to conduct research in the field of KOA, as well as highlight deficiencies and potential considerations in application in clinical practice. Finally, we highlight the diagnostic value of deep learning for future computer-aided diagnostic applications to complete this review.

## 1. Introduction

KOA is the most common form of arthritis and cause of activity limitation and physical disability in older adults [1]. Clinically, KOA is characterized by the gradual wear down of the protective cartilage that cushions the ends of the bones, and structural changes in joint tissues, including twisted bone and cartilage [2]. The main symptoms of knee OA include osteophyte formation, joint space narrowing (JSN), and subchondral sclerosis. In addition, pain is the main symptom of KOA, which drives patients to seek medical treatment and reduces the quality of life [3].

Moreover, KOA appears mostly in people over 55 years of age, with a higher prevalence in people over 65 years of age [4,5]. Indeed, according to global population research, it is considered one of the leading causes of disability, affecting 3.8 million people worldwide [6]. Furthermore, researchers estimate that at least 130 million people will suffer from KOA by the year 2050, along with the rising global number of aging people [7]. Nonetheless, early detection and treatment can help reduce KOA progression in the elderly and enhance their quality of life.

Furthermore, based on the literature, there is an urgent need for clinical tools that are will be able to diagnose and potentially predict KOA in relation to the recognized clinical and biological heterogeneity of KOA. Because of the rising incidence of KOA and its impact on functional limitations, health-related quality of life, health-care consumption, and total joint arthroplasty, clinical and scientific techniques that may accurately identify KOA early in its development are in high demand. The diagnosis of knee KOA is currently based on reported patient symptoms and X-ray imagery [8]. Additionally, various techniques are available for more advanced imaging modalities such as CT and MRI, which are associated with the 3D structure of the knee joint. However, these models are only accessible at large medical facilities, and the cost of the scan renders it unsuitable for regular KOA diagnosis. Therefore, osteoarthritis imaging by radiography remains the gold standard for KOA screening due to the low acquisition, safety, cost-effectiveness, speed, and wide accessibility.

**Figure 1:**
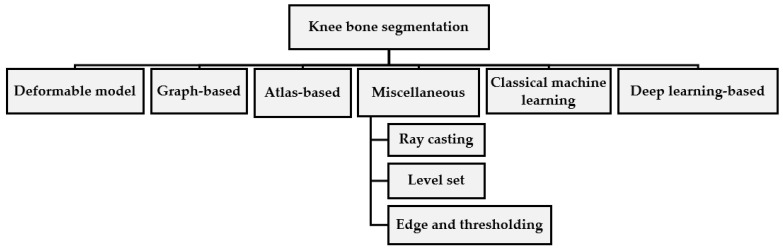
Methods of classifying the knee bones [12].

In addition, there are techniques of segmentation in knee joint research and clinical practice which have an important role in KOA diagnosis and classification [9]. Especially, machine learning and deep learning approaches have been extensively used in medical imaging to address problems of classification, detection, and related issues without the involvement of a radiologist [10,11]. Figure 1 presents the knee bone taxonomy methods.

Therefore, our study has the following contributions, which are summarized as follows:

- This paper provides a comprehensive survey and analysis of a wide range of state-of-the-art recent methodologies for knee bone segmentation. Moreover, we present quantitative results and the findings of other studies, in order to evaluate their potential and limitations;
- We perform an extended analysis of knee bone segmentation methods, taking it to the next level of depth by breaking the approaches down into their building pieces and emphasizing the algorithmic aspects;
- Unlike other studies, we not only investigate the existing methods, but also provide recommendations and future directions to enhance them;
- Finally, we highlight deep learning’s diagnostic value as the key to future computer-aided diagnosis applications to conclude this review.

The rest of this paper is divided into four sections. Section 2 presents knee bone segmentation techniques, Section 3 presents the approaches that have been used to construct this review, Section 4 provides discussion and recommendations, and Section 5 describes the conclusion.

## 2. Knee Bone Segmentation

**Figure 2:**
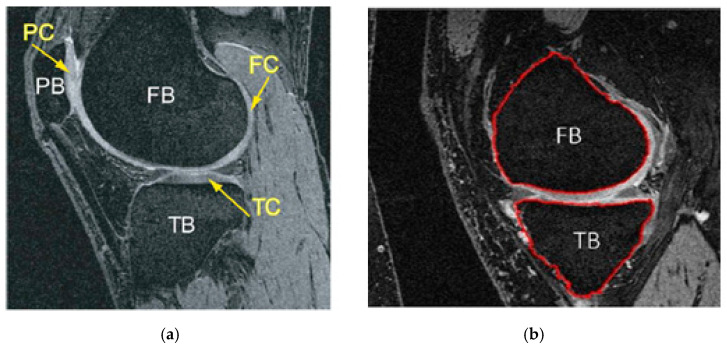
Knee bone segmentation has benefits over other tissues because of its location and anatomical size. (**a**) Illustrates the MR image of a knee joint—patella, femur, and tibia bones, readily apparent with the accompanying cartilage surfaces. TB = tibia bone, FB = femoral bone, PC = patellar cartilage, FC = femoral cartilage, TC= tibia cartilage. (**b**) Shows segmented tibia (TB) and femur (FB), which usually have better demarcation [19].

Osteoarthritis of the knee results in a constant loss of mineralization, causing its sensitivity to structural deformation [13]. Some structural changes can be seen on X-rays, including bone marrow lesions (BMLs), subchondral bone attrition (SBA), and osteoblasts, which are some of the radiologically apparent indicators of OA-related clinical studies. According to a study reported by (Hunter et al., 2006), subchondral BMLs are more evident across knee regions with increased biomechanical loading [14]. In contrast, other studies showed that the development of BMLs was associated with the loss of cartilage [13,15,16]. So, bone segmentation will be required for the detection and characterization of these biomarkers. Consequently, the following applications represent the goals of segmentation of the knee bone: firstly, to compute a bone model to investigate the effect of biomechanical stress at different localized knee sites; secondly, to quantify and monitor the changes of bone shape and surface associated with structural deformations; and finally, to produce a bone–cartilage interface (BCI) in order to extract cartilage tissue from the bone surface [15,17,18,19]. Figure 2 shows an example of a 2D bone structure and segmentation result.

Furthermore, prior knowledge of knee joint anatomy and completely automated segmentation algorithms are required for accurate bone surface detection. In the following subsection, a complete list of knee bone segmentation models is provided, from traditional models to deep learning.

### 2.1. Deformable Model-Based

Deformable models are semi-automated and extensively utilized approaches in clinical applications [20]. The mathematical basis of deformable models is an intersection of geometry, physics, and theory of approval. Geometry helps to describe the shape of objects; physics constrains the way the shape may vary across time and space and the theory of optimum approximation gives the formal basis on which the models adapt to observed data. Moreover, deformable models are different in terms of developing curves and representations of surfaces, for instance, geometric deformable models represent implicitly evolving curves and surfaces as a function of level set, while parametric deformable models explicitly represent curves and surfaces in their parametric form as energy reduction formulations and dynamic force formulations [21].

Furthermore, a primary deformable model was expanded using a previous shape information. Some notable expansions are as follows: active shape models (ASMs) [22], statistical shape models (SSMs) [23], and active appearance models (AAMs) [24]. Concretely, these deformable models generally need training in order to get information on the shape variability or appearance of the target item. Manual interaction allows the integration of previous information, such as a collection of landmarks to build a point distribution model (PDM). However, the most attractive deformable model nowadays is commonly called “snakes”, where “deformable contour models” or “Snakes” represent a particular instance of the general multidimensional deformable model theory [21,25]. Snakes are planar outlines that are helpful for several image analysis purposes, e.g., they are commonly used to estimate object frontier positions and forms in pictures based on the plausible assumption that borders are partly continuous or smooth [26]

For instance, in the case of using an active shape model for segmenting an image, the following steps should be employed [27]:

**Step 1**: Fitting a model to an image is possible given a rough approximation, and a model can be applied to an image. By selecting a set of shape parameters, we can determine, **b** the shape of the object in an object-centered coordinate frame;

**Step 2**: By using Equation (1), we can define the position, orientation, and scale of an instance A of the model in the image frame.

(1) $$A=T_{A_{t},Y_{t},s,o}\left(\bar{A}+\mathrm{Pb}\right)$$

A Euclidean transformation defines the position (A_t_, Y_t_), oriented (o), and scale (s) of the model as it appears in the image; and P represents the corresponding point in the rotated space. The following is an iterative approach to improving the fit between an instance, A, and an image:
**Active Shape Model Algorithm**Finding the closest nearby match for the point A′_i_ by analyzing a region of the image around each point A_i_.The parameters (A_t_, Y_t_, s, o, b) should be updated to fit the newly discovered points A.Set constraints on the parameters, b, to ensure plausible shapes (e.g., limit so [b_i_] < 3 $\sqrt{\lambda i}$).Continue until convergence is reached.

**Figure 3:**
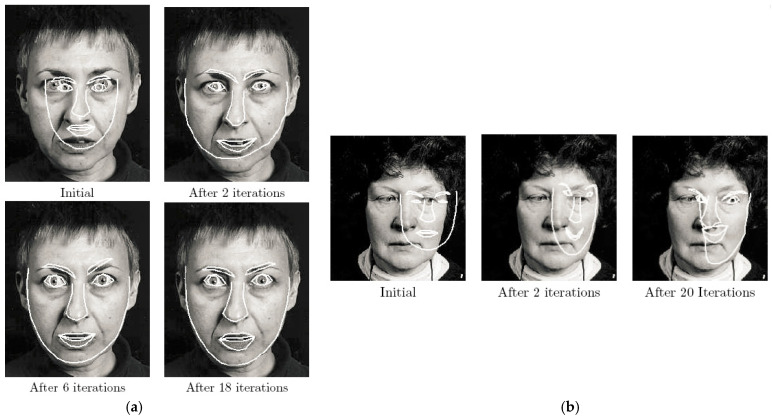
Illustrates the search with ASM for the face. (**a**) In the case of a point being near the target; (**b**) shows how the ASM can break down if the starting position is too far from the target [27].

Figure 3 demonstrates the search with ASM for the face in two cases. Moreover, the deformable model has been widely utilized with knee bone segmentation because of its consistency in form and size benefit. In addition, these models attempt to recognize low-level image data, such as borders and intensity areas, wherein MRI these characteristics are not easily recognizable and the complete segmentation of images may be defective [27,28]. These deformable models contain the following: SSM [17], active contour model (ACM) [29,30], and AAM [31].

In 2007, Fripp et al. [32] presented a study that represented a significant step towards the automated precise segmentation of cartilage; specifically, the automatic segmentation of bones and bone–cartilage interface (BCI) extraction in the knee. The segmentation was performed utilizing 3D ASM, which was initialized through a precise atlas registration. Then, the BCI was extracted from image information and prior knowledge about the probability that each point was part of the interface. Moreover, the patella surfaces, tibia, and femoral bones were taken from the database for manually segmented images, using the surface extraction technique of shrink-wrap. In addition, the proposed method used 2562 points, 10,242 points, and 10,242 points, correspondingly. Moreover, those surfaces were then utilized to create SSMs for each knee bone. The propagating surface’s pose and shape parameters were then trained to predict pose and shape variation inside the SSM. On the other hand, in 2010, Vincent et al. [33] proposed a complete automated model-based approach that divided bone and cartilage in the MR images of the knee. Besides, the presented segmentation approach was based on AAM from the Osteoarthritis Initiative data source of hand-selected samples. Moreover, the model was based on 3D DESS Sagittal water excitement images of the OAI database (available at https://oai.ucsf.edu/ (Date Accessed 21 July 2021) for public access). They utilized 80 individuals for the model from this dataset. The following are the number of correspondence points generated by this process: femur 60,457, tibia 39,239, femoral cartilage 37,249, and tibial cartilage 20,459. The results without adjusting to the protocol of the grand challenge were highly promising, and showed the strength of the system.

A similar concept to Fripp et al. [32] was found of automated bone segmentation through using the AAM model in 2010, by Seim et al. [34], where SSMs were produced for the tibia and femur. The SSM can provide a strong bone model, and was often used to remove BCI from the bone’s surface, in addition to forecasting the occurrence of radiological KOA. In addition, in 2011, Bindernagel et al. [35] introduced an articulated statistical shape model (ASSM) of the human knee. The model included the fluctuation of statistical form and the explicit freedom of the physiological joint movements model. Moreover, they presented a model for a knee joint segmentation technique based on medical image data. The capability of the model was evaluated via a collection of 40 clinical MRI data sets with accessible manual expert segments. Furthermore, in 2013, Neogi et al. [15] trained the AAM with 96 knees to understand the changes in the form and gray levels of femur, tibia, and patella texture. These data were then encoded as main components. Moreover, the AAM models were developed using a total of 69, 66, and 59 main components for the femur, tibia, and patella bone, respectively. Typically, deformable models provide robustness to both image noise and boundary gaps, and allow boundary elements to be combined into a coherent and consistent mathematical description. In this context, deformable models, particularly extension techniques, are capable of segmenting the knee joint [36].

### 2.2. Graph-Based Methods

The term “graph-based segmentation methods” refers to a group of algorithms in which pixels or voxels in an image sequence, as well as the relationships between them, are represented as a weighted undirected graph. Suppose G = (V, E) be a graph, where the pixel is represented as a node, v € V, and the interaction between two surrounding nodes is represented as edge, e € E ⊊ V × V [37]. Every edge is assigned by weight [38]. For instance, the edge between the nodes v_A_ and v_B_ has the associated weight w (v_A_, v_B_), reflecting a similarity measure between the nodes. The similarity criterion is calculated from color, texture, spatial distribution, intensity, hue, or any other characteristic between two vertices [39]. Retroactively, after the normalized cut, graph segmentation achieves more attention. Therefore, in this context, the graph separation is called a “cut”. A typical binary graph segment divides the graph into two subsections, i.e., *G_m_* and *G_n_*, where *G_m_* ∪ *G_n_ = V* and *G_m_ ∩ G_n_* = ∅, by reducing the degree of difference between *G_m_* and *G_n_*. We calculate the difference as the weight of the deleted edges:

(2) $$Cut\left(G_{m},G_{n}\right)=\underset{p\in G_{m},q\in G_{n}}{\sum }w\left(p,q\right)$$

where *G_m_* and *G_n_* are vertices in two separate subgraphs, and the total weights of the edges are called cut. Reducing this cut makes subgraphs different. However, cutting the graph into sub-graphs in a perfect way is not easy. Thus, a possible solution to this problem is to minimize the cut in Equation (2) through optimization methods, where a comprehensive review of the graph-based segmentation method is provided in [40,41].

**Figure 4:**
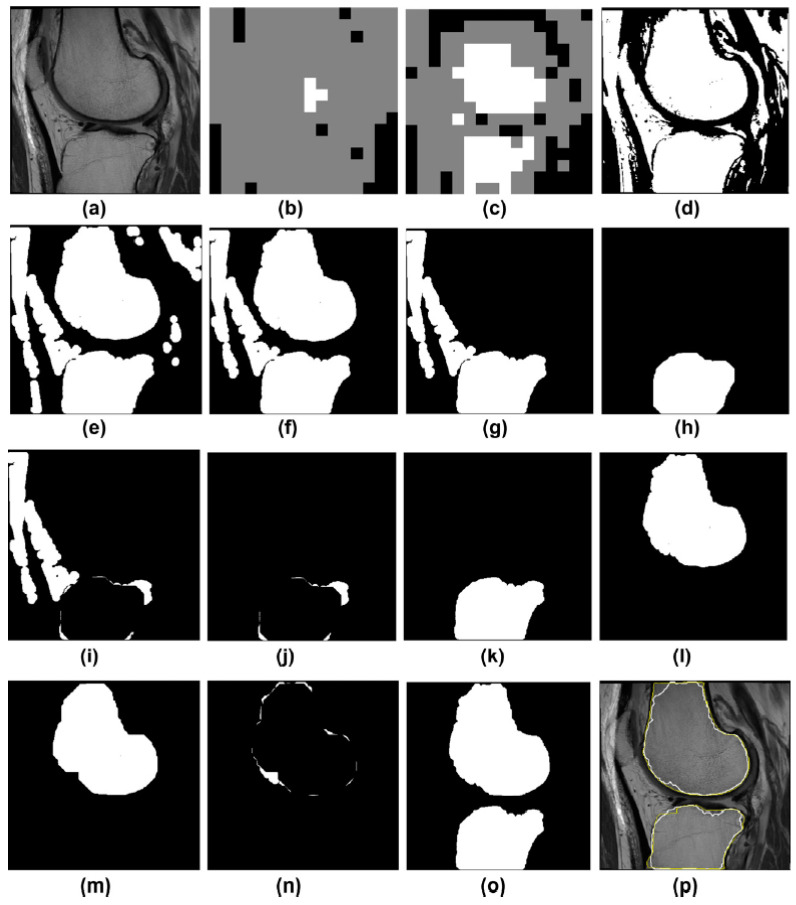
An example illustrates both ROI detection failures/recoveries and leak detection and correction. (**a**) An MRI image; (**b**) the ROI block is detected with only the femur bone detected, not the tibia; (**c**) after lowering the ROI detection threshold, both bones are detected; (**d**) mask for the GC output; (**e**) after morphological processes; (**f**) the resulting two potential skeletons, with a leak seen in the tibia bone; (**g**) the tibia bone has a leak that connects fat and other tissues to the tibia; (**h**) initial step in detecting a leak is to use a morphological opening; (**i**) residual content resulting from subtracting (**h**) from (**g**). (**j**) Following an examination of the remains in (**i**), the leak detection method identifies a leak and decides that only the pixels in the leak are affected; (**j**) are relevant to the tibia (**k**) after adding the appropriate pixels in (**j**) to (**h**), resulting in a leak-free tibia (**i**), the femoral mask (**l**) and (**m**). After applying the morphological aperture to check for leakage (**n**) the remaining pixels after subtracting (**m**) from (**i**). On this basis, it is concluded that there is no leak, and the pixels are reinserted (**o**). (**o**) femur and tibia masks as a result (**p**) GC segmentation in white and manual segmentation in yellow determined with DICE = 0.95 and 0.96 resolution for femur and tibia bones, respectively [45].

In 1993, Wu and Leahy [42] implemented graph cuts (gcuts) for image segmentation and developed a cost function, i.e., a minimal reduction, as shown in Equation (1), to frequently search for every possible piece, splitting the two knots at the lowest cost to achieve the optimal solution. However, the algorithm was biased to split a small part of the node. So, Jianbo and Malik [43] suggested a normalized reduction to tackle this issue. Boykov and Jolly also proposed graph cuts (Gcuts) in 2001. Graph cuts (gcuts) can be used interactively or automatically for image segmentation algorithms. Interactive gcuts are widely used to segment biological images, which include past user information into the local and border characteristics of images. Therefore, various studies [19,44,45,46,47] have performed gcuts for extracting the knee bone from the MR image. Furthermore, a content-based refinement operation is used to improve the segmentation output of the GC algorithm. Figure 4 illustrates an example of this operation [45]. Yin et al. [19] introduced a new method for simultaneous segmentation of many interactive surfaces belonging to multiple interacting objects, called LOGISMOS (Optimized Layered Graph Image Segmentation of Multiple Objects and Surfaces). The technique was based on algorithmic inclusion in a single n-dimensional graph of various spatial interrelationships, and followed by graph optimizations that resulted in a globally optimized s solution. In addition, the usefulness and performance of the LOGISMOS technique were proven for the segmentation of bone and cartilage. Although this system was trained on only a few examples, it reached a good performance. In addition, the approach for the simultaneous segmentation of the bone and cartilage consists of three steps:

- Pre-segmentation of bones;
- Mesh generation and optimization by Gcuts;
- Co-segmentation of knee bone and cartilage surfaces.

In the LOGISMOS framework, the optimum surface segmentation was defined in order to find a net surface of a guided graph with a minimal weight cost for every node. Furthermore, Park et al. (2009), and Ababneh et al. (2011) have suggested segmentation models for automated graph cuts, and the additional previous information was necessary in order to substitute manual seed. Ababneh et al. [45] proposed a novel automated knee bone segmentation system (MRI) which included a content-based two-pass discrete block discovery mechanism; also, it was designed to support automation, post-processing, and segmentation initialization. The proposed method was implemented as follows: block detection by categorizing the content of the image according to its similarity to the categories in training information gathered from the usual structures of the bone. Then, categorized blocks were utilized to construct an efficient divisional algorithm based on graphs. The result showed that the proposed segmentation approach did not need any interaction with users and could differentiate bone from extremely similar surrounding components such as high-precision adipose tissue.

Park et al. [44] introduced a fully automated method for the segmentation of bone chambers on the (MR) images of knee joints. The suggested technique efficiently used pre-segmented data for both form and intensity through using branch and mincut in an iterative manner to a limited subset of form templates configurations. Moreover, the optimum in each iteration was dissected and individually calculated between the whole sub-set of translation, rotation, and scale parameters, and movement was gently picked with the least amount of energy. Experimental results showed the enhanced accuracy and efficiency compared to when branch and mincut were applied to the whole range of parameters at once and when only shape priors were applied, respectively. Sufyan et al. [46] presented a novelty method for segmentation without the requirement of any user input, using efficient content characteristics based on graph cuts. Experimental findings on real MR images in the knee showed the efficient use of the Zijdenbos similarity indices of 95% of the scheme with average accuracy. Somasundar et al. [47] presented a graphic cut technique for segmenting the tibia and femoral bone from MRI/CT knee images. The proposed method used a median filter for the removal of noise first able. Then, the 3D model of a tibia and femoral bone was generated with segmented pictures for volume rendering. Finally, the 3D model of the tibia femoral bone was terminated in order to generate meshed elements. They concluded that this model may be used for analysis and pre-operative knee joint planning. Moreover, conventional semiautomatic graph cutting is mostly dependent on seeds that start and refine segmentation leading to considerable manual intervention. However, the DSCs 0.958 [44], 0.941 [45], and Zijdenbos Similarity Index (ZSI) 0.95 [46] are enhanced automated graph cut models.

### 2.3. Atlas-Based Methods

The atlases act as a regulator of shape allowing deformations within a reasonable range of variance derived from molds of anatomical shape, as well as appearance. Atlas includes three essential steps for segmentation (registration, selection, and propagation). Rohlfing et al. (2005) defined atlas as “incorporates prior anatomical data (i.e., locations and shapes of an anatomical structure), and distinguishes spatial relationships to other anatomical structures”. Therefore, the atlas techniques are designed to identify anatomical structures by mapping the coordinates of a specific image to a pre-constructed atlas. This step is called the registration process, which assigns the label of each voxel image to the appropriate label in the atlas by searching the label of its structure. For instance, in Equation (3), I refer to the correspondence between an image and an atlas referring to A. Where T refers to coordinate transformation that translates any specified image coordinates and ¥ describes the domain of I onto the atlas, A [48]. The mapping is as follows:

I(¥)→A(T(¥))(3)

In general, there are four ways to choose an atlas:

- Single atlas: Utilizes a separate segmented image; also, the selection might be random or based on particular criteria, for example, image quality;
- Probabilistic atlas (average shape atlas): Plots all of the original individual images on a common reference to produce a median image. Then, the original images are correlated with the first average to produce a new average. The mapping process occurs frequently until convergence;
- Best atlas: Used to determine the optimal segmentation from the results of the different atlases; one can check the similarity of the image using standardized mutual information and the size of the distortion after registration.
- Multiple atlases: This method applies various atlases to a raw image. Then, the segments are combined into a final hash based on the merging of the “voting rule” decision. This method applies various atlases to a raw image. Then, the segments are combined into a final hash based on the merging of the “voting rule” decision. This can be implemented as labeling cost C in Equation (4) per label l in {FB; BG. TB} (“FB”,”BG” and “TB” stand for femur, background and tibia bone in succession) is determined by the probability of recording each of the labels given image I in voxel Y site:

(4) $$E\left(u\right)=\underset{D}{\int }g\left|\left|\nabla Y^{u}\right|\right|+C\left|\nabla l^{u}\right|\mathrm{dYdl},D=Ω\times \varepsilon ,u\in \left[0,1\right],u\left(Y,0\right)=0,\left(Y,L\right)=1$$

(5) $$C\left(Y,l\right)=-\log\left(P\left(l|I\left(Y\right)\right)\right)=-\log\left(\frac{P\left(I\left(Y\right)|l\right).P\left(l\right)}{P\left(I\left(Y\right)\right)}\right),$$

where u refers to the multi-label image and $\nabla Y^{u}$ is the spatial gradient of u, $\;\nabla Y^{u}$=${\left(\frac{\partial u}{\partial Y},\frac{\partial u}{\partial Y},\frac{\partial u}{\partial Z}\;\right)}^{T}$ and $\nabla l^{u}$ is the gradient in the direction of the label, $\nabla l^{u}$=$\frac{\partial u}{\partial l};g$ controls the normalization of the properties and sets C labeling cost. Moreover, it is important to note that the background label “BG” is placed in order between the femur label “FB” and the tibia label “TB”, in order to achieve a symmetric formulation. The probability conditions P(I(Y)|FB) and P(I(Y)|TB) are calculated from the intensity of the image. Since bones appear dark in T1-weighted MR images, we assume a simple Equation (6) to estimate bone probabilities:

(6) P(I(Y)|FB)=P(I(Y)|TB)=exp(−βI(Y))

where β is set to 0.02 in implementation assuming I(Y)∈[0,100]. To compute the previous terms P(FB)and P(TB) in Equation (5), we use a multi-atlas registration technique followed by label fusion. Assume we have M atlases $A_{j}$ and their bone segmentations $S_{j}^{\mathrm{FB}}$ and $S_{j}^{\mathrm{TB}}$, where (j=1,2⋯M). The registration from an atlas $A_{j}$ to a query picture I is an affine registration $T_{j}^{\mathrm{affline}}$ followed by a B-Spline registration $T_{j}^{\mathrm{bSpline}}$. A spatial prior of femur and tibia for the query image is obtained by averaging all M propagated atlas labels.

(7) $$P\left(\mathrm{FB}\right)=\frac{1}{M}\sum_{j=1}^{M}(T_{j}^{\mathrm{bSpline}}{}^{\circ}\;T_{j}^{\mathrm{affline}}{}^{\circ}S_{j}^{\mathrm{FB}})$$

(8) $$P\left(\mathrm{FB}\right)=\frac{1}{M}\sum_{j=1}^{M}(T_{j}^{\mathrm{bSpline}}{}^{\circ}\;T_{j}^{\mathrm{affline}}{}^{\circ}S_{j}^{\mathrm{TB}})$$

**Figure 5:**
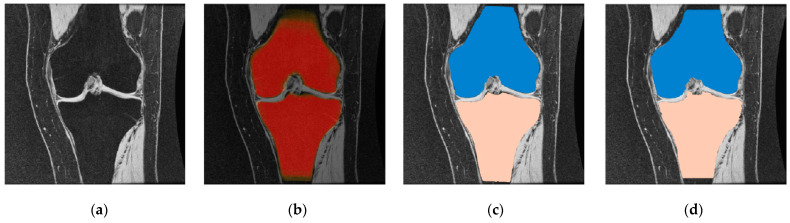
One sample slice’s bone segmentation in coronal view. (**a**) Original image; (**b**) multi-atlas-based spatial prior; (**c**) segmentation result; (**d**) expert segmentation [49].

After computing the spatial priors and local likelihoods, we integrate them into Equation (5) and solve Equation (4) to get the three-label bone segmentation. The bone segmentation will aid in the location of the cartilage in atlas-based cartilage segmentation [49]. Figure 5 shows an overview of the process of bone segmentation using a multi-atlas.

Several groups of study have used several templates for segmenting the knee based on atlas such as (Lee et al., 2014; Dam et al., 2015). In 2014, Lee et al. [50] developed a completely automated approach for segmenting knee magnetic resonance cartilage (MR) images, and assessed the method performance through a public open dataset. The presented segmentation system comprised three procedures: multi-atlas construction, implementing local weighted voting (LWV), and regional adaptation. All training instances were recorded in a goal image using a non-astringent registration system and the best matching atlases were picked for the atlas construction process. The result shows that it avoided the low precision caused by magnetic field inhomogeneity. Dam et al. [51] introduced a fully automated segmentation framework for knee MRI; the frame combined a rigid multi-atlas registry before the KNN-based classification of cartilage voxels and was manually trained in different bone, meniscal, and cartilage combinations. Validation comprised high- and low-field knee MRI cohorts from the center including the osteoarthritis initiative (OAI), and knee segmentation (SK I10). Empirical results were equivalent and equal to or better than previously reported automatic approaches to the manual radiologist segmentation.

### 2.4. Miscellaneous Segmentation Approaches

These approaches include additional models for knee bone segmentation involving ray casting, level set, edge and thresholding, and region growing.

Region growing is a method of segmenting anatomical structures that involves two key concepts: a seed voxel point located within the structure to be segmented, and a range of probable voxel grey-scale intensity levels that the region can achieve. Thus, the following steps are required to implement region growing in an image. In the set of definite areas $R_{t},n$ = 1, 2, 3, …, m, the low degree segmentation must require the given properties:

1. The region r in Y_j_ is connected to the region Y_i_ if there exists a sequence (Y_j_ … Y_i_). for instance, Y_t_ and Y_t+1_ are connected to R;
2. R is a continuous region if x and R are connected;
3. Whole image,

(9) $$(I)=\cup_{t=1}^{m}R_{t}$$

4. Equation (10)

(10) $$R_{j}\cap R_{i}=\emptyset \;\mathrm{for}\;j\neq i$$

**Figure 6:**
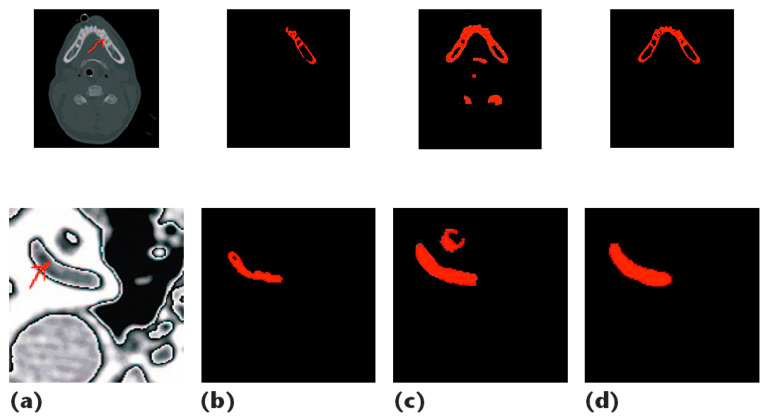
Analysis of the results of a general region-growing algorithm: (**a**) Original image (red arrow points to things (teeth and auris) to be segmented); (**b**) segmentation results from region growing using parameters r = 30; (**c**) results obtained using the robust split-and-merge algorithm; (**d**) the results showed that the edges of the images are more exact and smoother [53].

If these conditions are met, H ($R_{t}$) is true for every t, whereas H ($R_{j}\cup R_{i}$) is false for j ≠ i. H denotes the homogeneity property, while R denotes the area. If H(R) is false, divide the region into sub-regions. If H ($R_{t1}$ $\cup \;R_{t2}\cup R_{t3}$) is true, then combine them into one area up to zero for further splitting. Figure 6 shows that the results of the general region-growing algorithm are not good because fixed parameters limit the algorithm’s ability to handle growing regions [52].

Lee and Chung [54] suggested multi-phase segmentation of the knee bone model that would improve the contrast of the bone edges and the extraction bone boundary information, using a sequence of edge detection, thresholding, and contrast enhancements. In order to accomplish the final segmentation, the information was included in the region growing algorithm. Then, 40 knees were used to assess the model; however, the results were not obvious. Dodin et al. [55] developed a fully automated bone segmentation method based on the ray casting approach from MR images. The proposed method relied on MR image breakdown into many surface layers to locate the limits of bones and the automatic fusion of numerous partial segmentation objects to achieve the final full bone segmentation. Moreover, validation analyses were performed on 161 MR images from patients with knee osteoarthritis and the DSC reported 0.94 for the femur and 0.92 for the tibia. Similarly, Gandhamal et al. (2017) and Dalvi et al. (2007) applied their knee bone segmentation by using level set models. In particular, the region growing method was utilized by Dalvi et al. [56] to segment the knee bone, and then the segmentation refinement used the set algorithm of the Laplacian level. The proposed method was verified by the measurement of specificity (Spec) and sensitivity (Sens) in two healthy people. Gandhamal et al. [57] suggested a fully automated approach for the segmentation of the subchondral bone from knee MR images. According to the framework proposed, the preprocessing steps consisted of the following: image contrast optimization and automatic seed point selection, which were performed on all knee MRI images in datasets, followed by bone area extraction, borderline leakage detection, and correction of boundaries. The performance of advanced technology was evaluated by measuring sensitivity, specificity, dice similarity coefficient (DSC), average surface distance (Avgd), and root mean squared distance (RMSD).

### 2.5. Machine Learning Based

**Figure 7:**
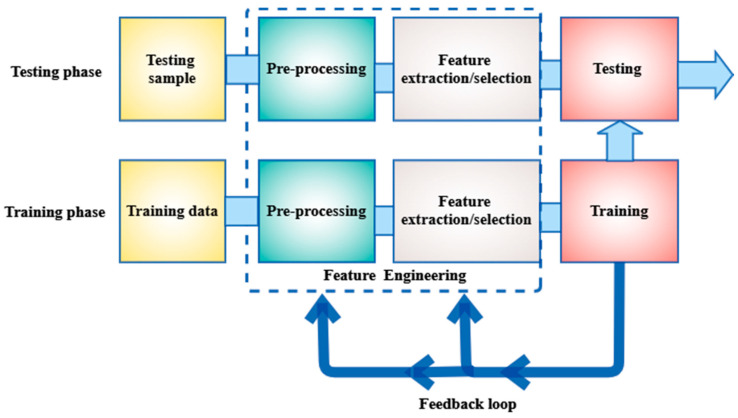
A typical system of machine learning [61].

Machine learning is the study of how computer algorithms (i.e., machines) may “learn” complicated connections or patterns from experimental data, resulting in (mathematical) models that link a large number of variables to target variables of interest [58]. As mentioned earlier, the ability to analyze complex cases with a huge amount of data and the maximum possible outcomes makes machine learning a valuable tool for KOA. It is noteworthy that machine learning (ML) has been applied in fields such as medicine, robotics, bioinformatics, biochemistry, meteorology, economics, agriculture, and economics. In 2019, the importance of implementing ML techniques to KOA was documented by Jamshidi et al. [59] and Klozyk and Matte [60]. The traditional systems of ML are applied in two phases, as shown in Figure 7: (1) cleaning of data to reduce noise, inconsistent examples, and missing data; (2) data integration where various sources of information are available; (3) data transformation includes normalization and discretization. The feature extraction/selection unit (also referred to as the feature engineering unit) attempts to generate and/or identify the most informative feature subset in which the learning model will be subsequently applied during the training phase [61].

The feedback loop enables changes to the pre-processing and feature extraction/selection units, which will help the learning model perform even better. During the testing phase, the trained model shows previously unseen samples (represented by images or trait vectors) that must be classified. Based on the characteristics contained in each sample, the model makes an appropriate decision (classification or regression). Moreover, the general machine learning framework includes data algorithms and prediction. Data is a set of observations used during training and testing, while a prediction algorithm learns metadata patterns to perform certain classification tasks. Classical machine learning employs a collection of hand-made discriminatory characteristics to characterize the object and to assign the most likely label image pixel to a classifier. In addition, the machinery learning family is large and includes supervised learning, semi-supervised learning, unsupervised learning, and reinforcement learning. Interestingly, supervised and unsupervised learning are the two major learning algorithms that may be used for machine learning.

Supervised learning examines the link between the input space x and the output label y, and it is most commonly applied to regression and classification problems. Common supervised learning algorithms include:

1. Decision Tree: The algorithm is structured like a tree, with branches and nodes. Each branch indicates the outcome, whereas each leaf node represents a class label. The method will sort characteristics in a hierarchical order from the root of the tree to the leaf node [62];
2. Naïve Bayes: The technique is based on the Bayes theorem, which assumes characteristics are statistically independent. The classification is based on the conditional likelihood that a result is produced from the probabilities imposed by the input variables [63];
3. Support Vector Machine: The algorithm aims to draw the most appropriate margins in which the distance between each category is maximized to the nearest margin. A margin is defined as the distance between two hyperplane support vectors. A bigger margin involves minor mistakes in categorization [64];
4. Ensemble Learning: A method of grouping multiple weak classifiers to build a strong classifier. It is known that aggregation methods can be used to improve prediction performance. Boosting and bagging are important ensemble learning techniques [65].

In unsupervised learning there are no labeled data. As a result, the unsupervised model infers from the input data based on similarity and redundancy reduction during training. Moreover, unsupervised learning is divided into two types: clustering and association rule. The following are some of the most often used unsupervised learning algorithms:

1. K-Means: This algorithm groups data into k-clusters based on their homogeneity, where the center of each cluster is an individual mean value. Moreover, the data values are allocated based on their closeness to the nearest average with the least possible error function during implementation [66];
2. Principal Component Analysis: This method aims to reduce the dimensionality of the data by finding a set of uncorrelated low dimensional linear data representations that have greater variance. This linear dimensional technique is useful for exploring the latent interaction of a variable in an unsupervised environment [67].

**Figure 8:**
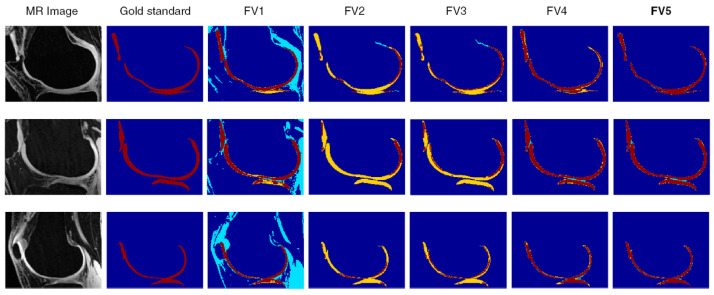
Segmentation findings for MR images using the hybrid SVM-DRF model with five types of feature vectors [68].

In the last decade, a new generation of frameworks has been introduced to solve challenges connected to knee joint segmentation utilizing learning-based methodologies or machine learning algorithms. The goal of the learning-based method is to find the features of each pixel $I_{j}$ from the data and assign a hash label ($l_{j}$ ∈ {1, 2, …, K}) to $I_{j}$. From the probabilistic situation, these learning-based methods predict a training set of labeled pixels by calculating the conditional probability P ($l_{j}|l_{j}$). Figure 8 demonstrates segmentation results for all three cartilage compartments using the joint support vector machine (SVM)-discriminative random field (DRF) model with FV1, FV2, FV3, FV4, and FV5. Feature vector 1 (FV1) is made up of four-dimensional normalized intensity data from multi-contrast MR images (from all four MR sequences); Feature vector 2 (FV2) consists of one-dimensional normalized intensity values of single-contrast MR images from the FS SPGR series and six-dimensional local image structure-based characteristics; Feature vector 3 (FV3) consists of 4 natural dimension intensity values of multi-contrast MR images and 24 dimension features based on the local image structure; Feature Vector 4 (FV4) consists of four natural dimension density values of multi-contrast and 3D MRI images of geometric information for multi-contrast MR images; Feature vector 5 (FV5) consists of 4 natural dimension intensity values of multi-contrast MR images, with a local 24 after features based on image structure and 3D geometry features of multi-contrast MR images.

Furthermore, several studies have used machine learning techniques for the diagnosis and predictions of knee osteoarthritis. Brahim et al. [69] presented a computer-aided diagnostic method for early knee osteoarthritis identification utilizing knee X-ray imaging and machine-learning algorithms. The proposed approaches were implemented as follows: first, preprocessing of the X-ray pictures in the Fourier domain was performed using a circular Fourier transform; then, MLR (multivariate linear regression) was used on the data to decrease the variability between patients with OA and healthy participants; for the feature extraction/selection stage, an independent component analysis (ICA) was used to reduce the dimensionality; finally, random forest and Naïve Bayes classifier were used for the classification task. Furthermore, the 1024 knee X-ray images from the public database Osteoarthritis Initiative were used to test this innovative image-based method (OAI). The results demonstrated that the suggested method had a high predictive classification rate for OA detection (accuracy of 82.98 percent, sensitivity of 87.15 percent, and specificity of up to 80.65 percent). Kubkaddi and Ravikumar [70] presented an automated diagnosis of knee osteoarthritis using a classifier based on support vector machines. Various textural and statistical characteristics were taken into account along with thickness when training the algorithm. The results showed that the SVM with RBF kernel, SVM with linear kernel, and SVM with the polynomial kernel were 95.45 percent, 95.45 percent, and 87.8 percent, respectively. In addition, Du et al. [71] studied to look for hidden biological information in knee MR images that could be used to predict osteoarthritis (OA). The presented study calculated the Cartilage Damage Index (CDI) information from 36 informative sites on the tibia and femoral cartilage compartment using 3D MR imaging and processed the feature set using PCA analysis.

Four machine learning methods (support vector machine (SVM), artificial neural network (ANN), Naïve Bayes, and random forest) were employed to predict the progression of OA, which was measured by the change of Kellgren and Lawrence (KL) grade, Joint Space Narrowing on Lateral compartment (JSL) grade, and Joint Space Narrowing on Medial compartment (JSM) grade. The findings of the experiments indicated that the medial feature set created a higher prediction performance than the lateral feature set and that the 36-dimensional total feature set generated the greatest prediction performance of all the feature sets. Kashyap et al. [72] developed a novelty method through hierarchical RF classifiers to learn the appearance of cartilage regions and their boundaries. The neighborhood approximation forest was used first to provide a contextual feature for a second-level RF classifier, which additionally analyzed local features and generated location-specific costs for the layered optimum graph image segmentation of multiple objects and surface (LOGISMOS) framework. The data were prepared using the just-enough interaction (JEI) approach, which provides fast and accurate post-processing. Halilaj et al. [73] presented a model for the longitudinal progression of KOA and built a prognostic tool that used data collected in one year to predict disease progression over eight years. The proposed model used a mixed-effects model and data of eight years from the Osteoporosis Initiative, specifically. Moreover, the presented method built LASSO regression models based on clinical data gathered within the first year to predict the likelihood of belonging to each cluster. Depending on the narrowing of the common space, topics were grouped as progressing or not progressing. In addition, based on pain scores, they were grouped as stable, improving, or getting worse.

### 2.6. Deep Learning-Based

**Figure 9:**
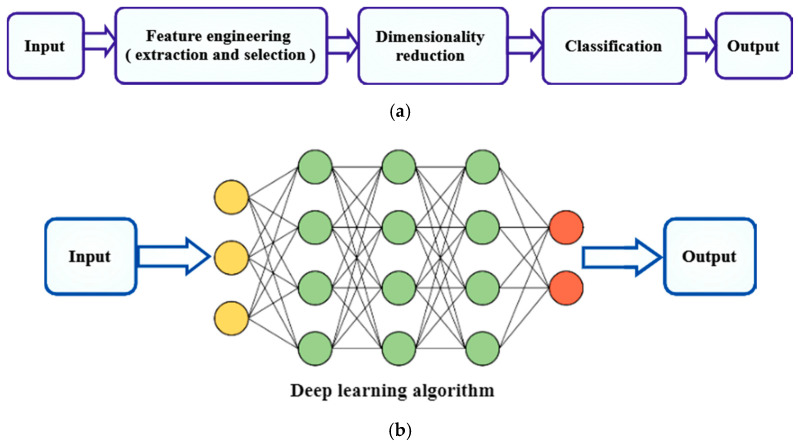
Knee bone segmentation using (**a**) classical machine learning and (**b**) deep learning. Classic machine learning feature architecture includes hand-picked representations and mapping, while deep learning uses multiple hidden layers to extract representations of hierarchical features [76].

Deep learning is a branch of machine learning that deals with algorithms inspired by the structure and function of the brain to create new architecture by transferring feature engineering (the process of converting raw data into features) onto the underlying learning system [74]. Moreover, this is a sophisticated machine learning model with automated hierarchical feature representation learning capability. Its general architecture consists of an input layer, hidden (feature extraction) layers, and an output (classification) layer [75]. From this standpoint, feature extraction and selection are discarded to achieve a completely trainable system that starts with raw or pre-processed input (e.g., image pixels or time-series) and ends with the final output of recognized objects or predicted values. Deep learning has recently received a lot of interest because of its huge analog power, ability for machine-learning characteristics, and best-in-class performance in handling challenging issues. Figure 9 shows a comparison between traditional machine learning and deep learning.

**Figure 10:**
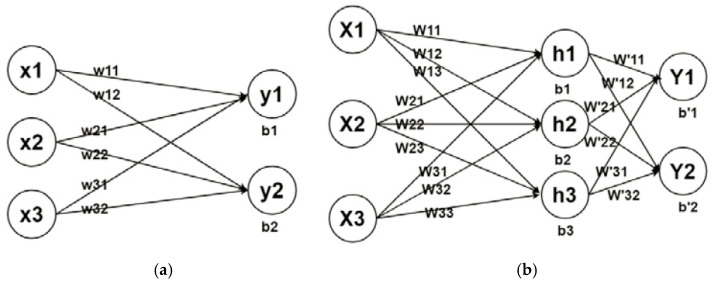
Explanation of the difference between a linear regression model and a simple learning model: (**a**) linear model regression model; (**b**) simplified deep learning model [77].

**Figure 11:**
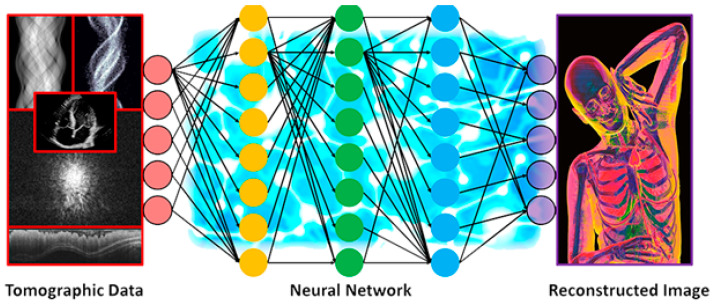
Process of segmenting medical images using deep learning [78].

Furthermore, deep NNs make use of deep architectures, expandable hidden modules, and nonlinear activation functions to model complex data, while one of their most attractive aspects is that they automate feature engineering, thus mitigating the need for domain expertise and powerful feature extraction. Deep learning uses many hidden layers to build nonlinearity map drawings. As simplified in Figure 10b, one hidden layer with three neurons is added and the nonlinear mapping between input and output is modeled by the following equations [77]. Moreover, Figure 11 describes the process of using deep learning for image segmentation [78].

(11) $$h_{1}=w_{11}\times x_{1}+w_{21}\times x_{2}+w_{31}\times x_{3}+b_{1}$$

(12) $$h_{2}=w_{12}\times x_{1}+w_{22}\times x_{2}+w_{32}\times x_{3}+b_{2}$$

(13) $$h_{3}=w_{13}\times x_{1}+w_{23}\times x_{2}+w_{33}\times x_{3}+b_{3}$$

(14) $$y_{1}=w_{11}^{\prime }\times h_{1}+w_{21}^{\prime }\times h_{2}+w_{31}^{\prime }\times h_{3}+b_{1}^{\prime }$$

(15) $$y_{2}=w_{12}^{\prime }\times h_{1}+w_{22}^{\prime }\times h_{2}+w_{32}^{\prime }\times h_{3}+b_{2}^{\prime }$$

The linear regression model is presented in Figure 10a. The input variables are three, and output variables are two, and they can be expressed by the following equations.

(16) $$y_{1}=w_{11}\times x_{1}+w_{21}\times x_{2}+w_{31}\times x_{3}+b_{1}$$

(17) $$y_{2}=w_{12}^{\prime }\times x_{1}+w_{22}\times x_{2}+w_{32}\times x_{3}+b_{2}$$

Currently, DL models have greatly improved the latest technology in many different sectors and industries, including healthcare [79]. DL forms can be moderated, partially supervised, or even unsupervised, where the major deep learning architectures are as follows: convolutional neural networks (CNN) are one of the most well-known deep learning (DL) networks, in which feature maps are extracted via image convolutions. A detailed overview of CNNs is given in [80]. Other typical constructs of DL architectures that belong to the family of undirected probabilistic graphical models are deep Boltzmann machines and deep belief networks. Recurrent neural networks (RNN) are another significant family of DL models, which establish unique topological connections between their neurons to encode temporal information in sequential input [81]. Moreover, auto-encoder is another unsupervised DNN whose basic concept is to encode high-dimensional data into a low-dimensional latent vector and then try to reconstruct the input data as perfectly as possible using just its coding [82]. During model training, the value of each node is evaluated by parameterizing weights using convolutional filters, and the objective function is then improved via backpropagation.

**Figure 12:**
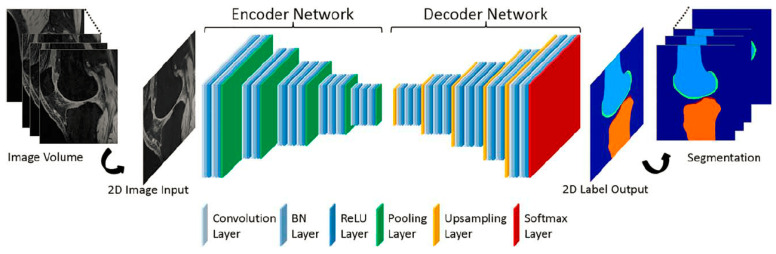
The SegNet CNN architecture is depicted in this diagram. SegNet is made up of two networks: an encoder and a decoder. This network’s final output is high-resolution pixel-by-pixel tissue categorization [83].

The following are some examples of deep learning-based knee bone segmentation for the early detection of osteoarthritis [83,84,85,86,87,88,89,90]. In general, CNN architecture is used in knee bone segmentation models, with minor changes. The notion of slice-wise segmentation was taken by [84] from [83]. Liu et al. [83] constructed a 10-layer SegNet framework and got rid of its completely connected layer after the decoder network, to perform semantic labeling on a two-dimensional knee image. The marching cube method was used to create a 3D simplex mesh using the processed labels. The simplex mesh was then transferred through a 3D simplex deformable process, where each segmentation object was fine-tuned individually depending on the original image. Figure 12 presents *Liu*’s method.

Ambellan et al. [84] presented a technique for automatically segmenting knee bones and cartilage using magnetic resonance imaging (MRI) that integrates anatomical form knowledge with convolutional neural networks (CNNs). The proposed technique included 3D Statistical Shape Models (SSMs) as well as 2D and 3D CNNs to accomplish the robust and accurate segmentation of even severely diseased knee components. Data from the Osteoarthritis Initiative (OAI) and the MICCAI grand challenge “Segmentation of Knee Images 2010” were used to train the shape models and neural networks (SKI10). The experimental setup was open to the public to advance research in the field of medical image segmentation. The result showed that integrating localized classification with CNNs and statistical anatomical information with SSMs yielded a cutting-edge segmentation technique for knee bones and cartilage using MRI data. Despite this, good performance was achieved to calculate huge computer resources and local training. For example, general-purpose graphics cards with smaller memory were not able to support 3D convolution, so it would not be easy to expand the model to process larger datasets without appropriate graphics cards. Moreover, the 3D model was trained on small subsections of 64 × 64 × 16 voxels along the osteoclastic lines to reduce the computational burden and compensate for the inability of SSM to provide osteoblast details. However, the choice of training compromised the voxel intensity and surrounding texture characteristics.

**Figure 13:**
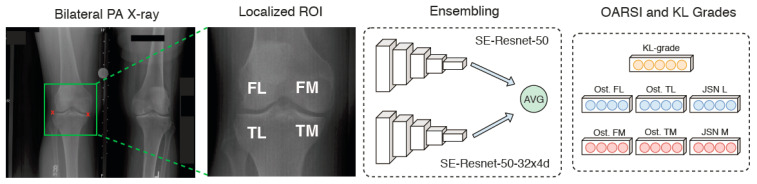
Schematic representation of the workflow of [87] approach.

In light of the aforementioned restrictions, Cheng et al. [85] introduced a simplified CNN model known as a holistically nested network (HNN) for femur and patella segmentation. HNN eliminates the decoding path to create a forward-feeding network, reducing the computational size of the graphic card. Furthermore, the network was trained on a complete knee picture using a 1 × 1 convolution at the first layer (to create fine features such as edges) up to a 32 × 32 convolution at the fifth layer (to produce coarse details such as bone structure); therefore, it learnt both local and global contextual information. Finally, a weighted fusion layer was created to average the probability map at each layer and compute the final prediction in a sequential manner. Although the authors attempted a complete validation against current state-of-the-art methods, they were hampered by the kind of bone selection (immature versus mature bone, and distinct bone compartment) and the lack of public ground truth. Furthermore, despite its superior resilience, deep learning model training is computationally intensive. Thus, according to Ambellan et al. (2019), implementing a deep learning model on 50,000 large-scale data images would take 43 weeks on a single computer node, emphasizing the significant cost of computation. Although some academics have reduced CNN design in order to minimize complexity, the problem still needs further investigation. On the other hand, there are a lot of models in deep learning that have been presented for the early detection of osteoarthritis, such as that by Lim et al. [86], who offered an automated osteoarthritis prediction by using a deep learning algorithm with a scaled PCA, based on medical usage and health behavior data (from 5749 patients) without any hand-crafted features, verified in a large population. A principal component analysis with quantile transformer scaling was used to identify osteoarthritis in the patients’ basic medical data. In addition, the proposed model was able to achieve an AUC of 76.8 percent while minimizing the effort required to create features. Moreover, they concluded that patients and physicians may use this method to prescreen for osteoarthritis and save money and time in the hospital. Tiulpin and Saarakkala [87] established an automated technique for predicting KL and OARSI grades from knee radiographs. The proposed approach was based on deep learning and employed a 50-layer ensemble of residual networks and applied ImageNet transfer learning with fine-tuning. The empirical result showed that cross-validation transfer learning was beneficial for automatic OARSI grading; however, that simultaneous prediction of KL and OARSI grades leads to poor performance. Figure 13 presents the introduced method.

Antony et al. [88] introduced a new technique to automatically evaluate the severity of KOA using X-ray images. The presented approaches implemented as follows, in order to calculate the severity of KOA automatically: firstly, they located the knee joints automatically; secondly, localized knee joint pictures were categorized. Furthermore, the introduced method used a fully convolutional neural network to recognize the knee joints automatically (FCN), and convolutional neural networks (CNN) were trained from scratch to automatically quantify the KOA severity optimizing a weighted ratio of two-loss functions: mean-squared loss and categorical cross-entropy. The benefit of this joint training was providing multi-class classification and regression outputs.

Both OAI and MOST datasets were utilized to test the proposed technique. The findings were highly encouraging and exceeded previous approaches. Tiulpin et al. [89] presented a new computer-aided diagnostic technique based on the deep Siamese convolutional neural network to automatically quantify KOA severity according to the Kellgren–Lawrence grading system. The proposed approach was trained only on data from the Multicenter Osteoarthritis Study, and it was verified on 3000 individuals (5960 knees) from the Osteoarthritis Initiative dataset. The empirical result demonstrated that emphasizing radiological characteristics influences network decisions. Such information makes the decision-making process more transparent for the practitioner, which increases trust in automated approaches. Furthermore, according to the annotations provided by a committee of clinical experts, the presented approach produced a quadratic Kappa coefficient of 0.83 and average multiclass accuracy of 66.71 percent. Tiulpin et al. [91] presented a multi-modal machine learning-based OA progression prediction model that takes into account raw radiography data, clinical exam results, and the patient’s past medical history. An independent test set of 3918 knee pictures from 2129 individuals was used to validate this method. The area under the ROC curve (AUC) for the proposed approach was 0.79 (0.78–0.81), and the average precision (AP) was 0.68 (0.66–0.70). Moreover, they mentioned that the proposed technique might assist in generating tailored treatment strategies by considerably improving the subject selection procedure for OA medication development studies. Christodoulou et al. [90] studied new efficient research through using deep neural networks as a novel machine learning technique for classification problems, taking into consideration a vast number of medical variables that influence OA. The suggested methodology’s potential was proven by categorizing distinct subgroups of control participants based on self-reported clinical data and assigning a knee OA diagnostic category. Moreover, age, gender, and obesity were used to divide the studies into subgroups. To validate the proposed deep learning approach, a comparative study between the proposed DNN and other benchmark machine learning techniques recommended for classification was performed, and the results revealed the efficiency of deep learning in the diagnosis of KOA. Furthermore, the majority of DL approaches used for musculoskeletal structural segmentation are 2D CNNs that use 2D convolutions on a sagittal orthogonal image in a slice-wise segmentation procedure [83]. The fundamental reason for this is that GPU memory is restricted, which means that 3D patch-based CNN techniques have limited spatial context.

## 3. Approaches

### 3.1. Research Approach to Literature

**Figure 14:**
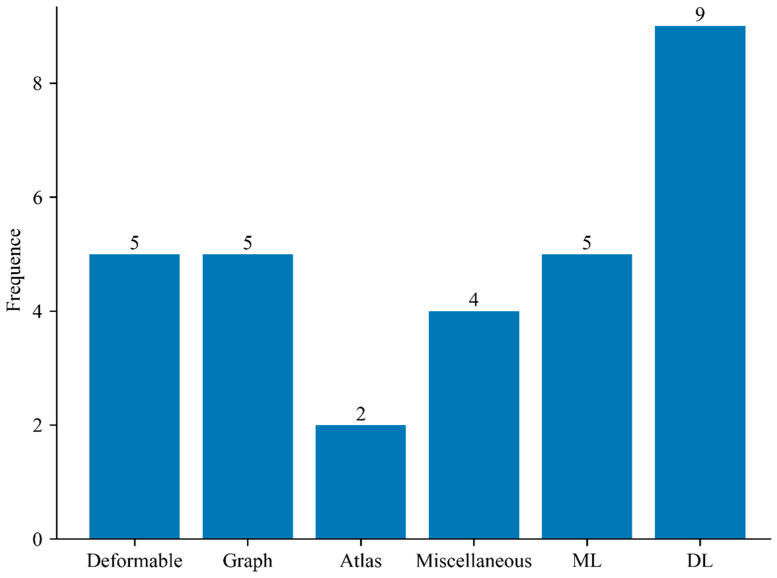
Demonstration of the number of papers reviewed for each method in KOA studies.

This survey was based on research publications found using the Google Scholar, PubMed, and Scopus search engines between 1991 and 2021. During our investigation, we identified articles that used segmentation techniques, machine learning, and deep learning to study KOA. In particular, the terms machine learning, deep learning, and knee osteoarthritis were used. The presence of one of the three terms indicated as keywords, either in the title or in the abstract of each article, was a requirement for inclusion in our study. Moreover, the bar chart in Figure 14 describes the distribution of the number of papers that were reviewed for each of the taxonomies of knee bone in KOA studies.

### 3.2. Estimated Results

The studies reported in this article can be divided into six categories, namely: (1) deformable models (5 studies), (2) graph-based models (5 studies), (3) classical machine learning techniques (5 studies), (4) miscellaneous (4 studies), (5) deep learning-based models (9 studies), and (6) atlas-based models (2 studies). Then, after separating the articles, the following information was extracted from each article: year of publication, author, region of interest, segmentation method, sequence type, data (X-ray, MRI, clinical data), feature engineering approaches, learning algorithm methods, validation, and empirical results (performance evaluation).

### 3.3. Data Sources

The majority of advanced analytical models to forecast knee osteoarthritis based on knee bone segmentation and knee articular cartilage morphology used imaging technology (either MRI or X-ray). Recently, the combination of multimodal data (medical images, clinical data) has formed the basis for more powerful and efficient models. OAI, SKI10, and MOST were the most frequently used databases to check the performance of the aforementioned hashing approach. Validation was performed using the LOOV, k-fold CV, random or expert manual assessment. An overview of all KOA fragmentation studies identified for our survey is presented in Table 1 and Table 2. Moreover, a variety of complex methods have been described to improve the quality of accessible raw data, or to overcome the dimensionality curse, including: (i) topological data analysis, ICA, PCA for dimensionality reduction; and (ii) CNN to extract new, more informative deep features for images.

## 4. Discussion and Recommendations

Our literature survey outlined several methods for creating segment knee bones in MR images, the current usage of machine learning methods in KOA diagnosis, and prediction challenges. In conclusion, there were five main points. First, segmentation of the knee bone can be performed by adopting various levels of automation, from manual to fully automated. Moreover, the development of a segmentation model based on MRI scans may lead to the adoption of hypothetical surgical procedures for planning real surgery, and the improvement of virtual surgery solutions could improve the patient’s anatomic structure. Second, unlike shape, atlas, graph, and machine learning approaches, segmentation models in this category were not dependent on any training dataset or user input. Instead, a number of preprocessing and image property learning processes were used to bridge the learning gap, ensuring the model remained automated. Third, to achieve the final bone segmentation based on updated image attributes, a variety of methodologies have been used. While these models were able to overcome basic anatomical aspects of bone, their applicability is be largely dependent on the tissue and image properties. In addition, some models require predetermined threshold values. Thus, it may be difficult to generalize these models to datasets of larger sizes compared to modern machine learning techniques, particularly deep learning. Four, the advancement of artificial intelligence technology has led to the emergence of new machine learning techniques in that can: (i) improve our understanding of the disease’s onset and development; (ii) offer new data-driven techniques that could help diagnose and forecast KOA in the early stages; (iii) play a crucial role in the direction of these models by extracting important knowledge from many types of clinical data (biomechanical parameters, pictures, and kinematics) and developing innovative solutions that incorporate data from as many different sources as feasible. This is inspired by the promising accuracy of the results shown by deep learning-based segmentation models. Deep learning has been applied to a wide range of computer-aided diagnosis applications, such as detection [83,86] and classification [88,91,92] in MRI and radiographs. Therefore, the main goal for these models is to diagnose and prevent knee OA at an early stage when cartilage deterioration is still reversible. Related applications for KOA include OA classification by radiographic such as [69,73,87,88,89], predicting knee pain by MRI [92], or using radiography to forecast OA progression [91]. Classification is the process of determining the likelihood of a label for a given input image using an algorithm. Furthermore, according to the literature, end-to-end deep neural network quantification of OA severity is critical for providing more precise computer-aided diagnoses to assist physicians in evaluating the severity of OA patients. Even before the implementation of deep learning, Shamir et al. [93] used an open-source classical machine learning software dedicated to biological image analysis, in order to categorize normal and diseased knee images. Moreover, Ashinsky et al. [94] used the weighted neighbor distance utilizing the compound hierarchy of algorithms representing the morphology (WND-CHRM) algorithm. Finally, the advantage of DL algorithms is that they automatically learn contextual information without requiring any high-computing spatial structure modeling, such as in model-based or atlas-based techniques, as this is computationally expensive. In this study, we reviewed different methods, focusing on the use of DL in knee articular bone segmentation. Nonetheless, the following recommendations and future research trends are suggested to offer appropriate methods for managing KOA:

- The development of a useful tool based on CNNs for assessing morphological and structural changes in the musculoskeletal system could be an interesting research field for assisting clinical applications, particularly for longitudinal assessments;
- More research is needed to improve current methods to address issues such as a lack of full assessment for intensity inhomogeneity and clinical practices;
- Combining DL strategies with other machine learning approaches such as KNN, SVM, and so on, can achieve an acceptable result;
- The design and development of a 3D CNN learning-based framework for a graph representation of knee joints that can accommodate both edge and shape information for the graph.

## 5. Conclusions

This survey provides six segmentation methods for KOA diagnosis, beginning with conventional methods such as a deformable, graph, miscellaneous, atlas, and state-of-the-art ML and DL, especially those that have been presented in the last few years. As the summary tables demonstrate, the comparison of methodologies is not an easy undertaking. The key obstacles were the lack of uniform databases and standardized benchmarks. Furthermore, due to varied testing datasets, comparing and evaluating the methodologies based on their published experimental outcomes was difficult. Consequently, these findings should be interpreted with caution, due to the fact that the segmentation method’s accuracy was highly dependent on the dataset. Therefore, we chose three publicly available datasets (MOST, SKI10, and OAI) with differing degrees of KOA severity to illustrate this issue. Knee joint segmentation approaches have been utilized alone or in combination with other procedures in a vast number of publications, and hybrid techniques have yielded positive outcomes [44,46,51,55,56,84]. As a result, learning-based methods can be combined with other methods to improve segmentation results. On the other hand, according to this survey, learning-based approaches dominate the field of knee bone segmentation. At present, artificial intelligence has transformed the direction of knee OA research towards prediction and early detection. Deep learning has proven considerable potential in terms of generalizability, robustness, and versatility, and innovative diagnostic apps are gradually becoming the state-of-the-art technology of the future. More research is needed to confirm the clinical application of deep learning technologies in order to meet future difficulties.

## Figures and Tables

**Table 1:** Summary of automatic knee bone segmentation based on deformable, graph, atlas, miscellaneous models.

Ref.	Year	Segmentation Technique	No. of Samples	Sequence Type	Region of Interest	Metric
[32]	2007	ASM-SSM	20 health samples	FS SPGR	Femur/Tibia /Patella	DSC: 0.96(FB); 0.96(TB) and 0.89 (PB)
[33]	2010	AAM	80 subjects	DESS	Femur/Tibia /Cartilage	AvgD:0.88 (±0.24) (FB); 0.74 (±0.21) (TB), RMSD: 1.49 (±0.44) (FB); 1.21 (±0.34) (TB) AvgD: 36.3 (±5.3) (FC); 34.6 (±7.9) (TC), RMSD: −25.2 (±10.1) (FC); −9.5 (±18.8) (TC)
[34]	2010	ASM-AAM	40 clinical MRI samples	T1 weighted SPGR	Femur/Tibia /Cartilage	AvgD:1.02 (±0.22) (FB); 0.84 (±0.19) (TB), RMSD: 1.54 (±0.30) (FB); 1.24 (±0.28) (TB) AvgD: 34.0 (±12.7) (FC); 29.2 (±8.6) (TC), RMSD: 7.7 (±19.2) (FC); −2.7 (±18.2) (TC)
[35]	2011	SSM	40 clinical samples	CTF	Femur/Tibia	For single-object (SSM) DICE: 0.94 (±0.02) (FB); 0.86 (±0.10) (TB)
[15]	2013	AAM	178 samples	Sagittal 3-D double-echo	Femur/Tibia /Patella	Odds ratio 12.5 [95% CI 4.0–39.3] for (K/L grade of 0) and [95% CI] 1.8–5.0, *p* < 0.0001 for OA after 12 months in patients in the lowest tertile grade compared to those in the top tertile grade.
[19]	2010	LOGISMOS	69 images	3D DESS WE	Femur/Tibia/Patella	DSC ± SD: 0.84 ± 0.04(FC);0.80 ± 0.04 (TC); 0.80 ± 0.04 (PC)
[45]	2011	Graph cuts	376 images	T2-weighted	Femur/Tibia	DSC: 0.936 (FB); 0.946 (TB); 0.941 (FB + TB)
[44]	2009	Graph cuts	8 images	DESS	Femur/Tibia/Patella	DSC: 0.961 (FB); 0.857 (PB); 0.970 (TB); 0.958
[46]	2010	Graph cuts	30 images	T2 sagittal map	Femur/Tibia	Zijdenbos Similarity Index (ZSI) for Avg 95%; Std 0.028; Median 0.96; Min 0.87; Max 0.98.
[47]	2020	Graph cuts	65 slices	T1 sequence	Femur/Tibia	Mean Square Error (MSE): 0.19
[50]	2014	Multi-atlas	100 training; 50 test	T1 weighted GRE FS	Femur/Tibia	ASD ± SD: 0.63 ± 0.17 mm (FB); 0.53 ± 0.25 mm (TB)
[51]	2015	Multi-atlas, KNN	The samples from CCBR OAI and SKI10 were used	T1 weighted Turbo 3D	Tibia	DSC ± SD (training): 0.975 ± 0.010 (TB)
[55]	2011	Ray casting	161 samples	GRE FS	Femur/Tibia	DSC ± SD: 0.94 ± 0.05 (FB);0.92 ± 0.07 (TB)
[56]	2007	Region growing; Level set	2 samples	T1 weighted	Femur/Tibia/Patella	Sens: 97.05% (FB); 96.95%(TB); 92.69% (PB) Spec: 98.79% (FB); 98.33%(TB);
[57]	2017	Level set; predefined Threshold	8 samples	DESS	Femur/Tibia	DSC ± SD: 90.28 ± 2.33% (FB); 91.35 ± 2.22% (TB)
[54]	2005	FLoG edge detector; Threshold; Wavelet transforms (WT)	40 samples	GE Signa Horizon LX 1.5 Tesla	Femur/Patella	The results show that the proposed method can segment the femur and patella robustly even under bad imaging conditions.

**Table 2:** Summary of deep learning and machine learning methods for studying knee bone segmentation and classification.

Ref	Year	Data	Dataset	Feature Engineering	Learning Algorithm	Validation	Results
[69]	2019	X-ray	OAI	ICA	Random forest; Naïve Bayes	Leave-One-Out (LOO)	87.15% sensitivity; 82.98% accuracy and up to 80.65% for specificity
[70]	2017	MRI	From hospital	GLCM	SVM with the linear kernel; SVM with RBF kernel; SVM with polynomial kernel	147 images training 66 images testing	95.45% accuracy; 95.45% accuracy; 87.8% accuracy
[71]	2018	MRI	OAI	PCA	SVM Random forest Naïve Bayes ANN	10-fold cross-validation	For JSL grade prediction the best performance was achieve for random forest AUC = 0.785 and F-measure = 0.743, while for the ANN with AUC = 0.695 and F-measure = 0.796.
[72]	2016	MRI	OAI	k-means clustering; Neighborhood approximation forests	LOGISMOS Forest Classifier Hierarchical Random	108 baseline MRIs and 54 patients’ 12-month follow-up scans	4D cartilage surface positioning errors (in millimeters)
[73]	2018	Pain scores and X-rays	OAI and MOST	PCA	LASSO regression	10-fold cross -validation	AUC of 0.86 for Radiographic progression
[83]	2018	MRI	SKI10	Not used	CNN	3D-FSE images and T2 maps	ASD ± SD: 0.56 ± 0.12 (FB); 0.50 ± 0.14 (TB)
[84]	2019	MRI	SKI10,OAI imorphics, OAI ZIB	Not used	2D/3D CNN and combination of (SSMs)	2-fold cross-validation	(i) 74.0 ± 7.7 total score. (ii) DSC: 89.4% (FC). (iii) DIC: 98.6% (FB), 98.5% (TB), 85.6% (TC), 89.9% (FC).
[85]	2020	MR	National Institutes of Health (NIH), SKI10	Not used	HNN deep learning	9-fold cross-validation	DSC ± SD: 0.972 ± 0.054 (FB); 0.947 ± 0.0113 (PB)
[86]	2019	X-ray	Korea Centers for Disease Control and Prevention (KCDCP)	PCA	Deep Neural Network (DNN)	(66%) train (34%) test, 5F-CV, (50%) train (50%) test	76.8% AUC
[87]	2020	X-ray	OAI,MOST	Not used	Ensemble and CNN	19,704 train 11,743 test	0.98 Average precision and 0.98 ROC
[88]	2017	X-ray	OAI,MOST	FCN	CNN	30% testing 70% training	Accuracy 60.3% for (multi-class Grades 0–4)
[89]	2018	X-ray	OAI,MOST	FCN	CNN ResNet-34	67% train, 11% validation, 22% testing	Accuracy 66.71% (multi-class Grades 0–4)
[91]	2019	Clinical data, X-ray	OAI,MOST	CNN	Gradient Boosting Machine (GBM) and Logistic Regression (LR)	MOST dataset for testing and OAI dataset for training, 5F-CV	Accuracy 0.79
[90]	2019	X-ray	OAI	Cascade	Deep Neural Network (DNN)	10-fold cross	82.98% Accuracy 87.15% Sensitivity 80.65% specificity

## Data Availability

Not applicable.
